# Cytotoxicity and Mutagenicity of Narrowband UVB to Mammalian Cells

**DOI:** 10.3390/genes11060646

**Published:** 2020-06-11

**Authors:** Dylan J. Buglewicz, Jacob T. Mussallem, Alexis H. Haskins, Cathy Su, Junko Maeda, Takamitsu A. Kato

**Affiliations:** Department of Environmental & Radiological Health Sciences, Colorado State University, 1618 Campus Delivery, Fort Collins, CO 80523, USA; dbuglewi@rams.colostate.edu (D.J.B.); jake.mussallem@gmail.com (J.T.M.); 2013ahaskins@gmail.com (A.H.H.); cathy50720@gmail.com (C.S.); junkorv0507@yahoo.co.jp (J.M.)

**Keywords:** narrowband UVB, broadband UVB, cytotoxicity, HPRT, SCE, DNA damage

## Abstract

Phototherapy using narrowband ultraviolet-B (NB-UVB) has been shown to be more effective than conventional broadband UVB (BB-UVB) in treating a variety of skin diseases. To assess the difference in carcinogenic potential between NB-UVB and BB-UVB, we investigated the cytotoxicity via colony formation assay, genotoxicity via sister chromatid exchange (SCE) assay, mutagenicity via hypoxanthine phosphoribosyltransferase (HPRT) mutation assay, as well as cyclobutane pyrimidine dimer (CPD) formation and reactive oxygen species (ROS) generation in Chinese hamster ovary (CHO) and their NER mutant cells. The radiation dose required to reduce survival to 10% (D_10_ value) demonstrated BB-UVB was 10 times more cytotoxic than NB-UVB, and revealed that NB-UVB also induces DNA damage repaired by nucleotide excision repair. We also found that BB-UVB more efficiently induced SCEs and HPRT mutations per absorbed energy dosage (J/m^2^) than NB-UVB. However, SCE and HPRT mutation frequencies were observed to rise in noncytotoxic dosages of NB-UVB exposure. BB-UVB and NB-UVB both produced a significant increase in CPD formation and ROS formation (*p* < 0.05); however, higher dosages were required for NB-UVB. These results suggest that NB-UVB is less cytotoxic and genotoxic than BB-UVB, but can still produce genotoxic effects even at noncytotoxic doses.

## 1. Introduction

As ultraviolet C (UVC) is blocked by the earth’s atmosphere, it is ultraviolet A (UVA) and ultraviolet B (UVB) radiation that are responsible for the effects of ultraviolet radiation (UVR) on the epidermal layer of the skin, but can also be used to treat skin diseases. Phototherapy utilizing artificial UVB exposure has become a valuable therapeutic tool in dermatology for treatment of skin diseases, such as psoriasis and vitiligo [1,2]. Unlike UVB, UVA is relatively ineffective for treatment of skin diseases unless used with the light-sensitizing medication psoralen, which is administered topically or orally. This process, called P-UVA, slows down excessive skin cell growth and can clear psoriasis symptoms for varying periods of time [3]. There are two types of UVB phototherapy treatments: broadband UVB (BB-UVB) and narrowband UVB (NB-UVB) [4]. The major difference between them is that BB-UVB composes the full spectrum of UVB wavelengths (280–315 nm), while NB-UVB light releases a smaller range of ultraviolet light (e.g., 311 nm monochromatic). NB-UVB has become favored over BB-UVB, as it has been demonstrated to be more effective in the treatment of psoriasis [2], as well as effective in treating polymorphic light eruption [5], atopic eczema [6], and many other inflammatory dermatoses. It is also considered safer and/or more practicable than P-UVA in the management of psoriasis [7].

These beneficial effects are believed to be the result of the ability of UVB to alter cell proliferation and induce immunosuppression. Treatment with UVB exposure can suppress the accelerated DNA synthesis in psoriatic epidermal cells, as it is known to cause a reduction in DNA synthesis [8]. UVB exposure also upregulates the tumor suppressor gene product p53, which is involved in control of the cell cycle. These factors are also important for apoptosis, which is seen in the skin after UVB exposure and for the prevention of skin carcinogenesis [9]. On the other hand, these factors can also lead to UVB being carcinogenic. UV light is known to directly induce cyclobutane pyrimidine dimers (CPDs), pyrimidine-pyrimidone (6-4) photoproducts [(6-4) PPs] and their related Dewar valence isomers and indirectly cause DNA damage through the production of reactive oxygen species (ROS) [10,11]. Direct absorption of UVB energy by nucleotides leads to DNA photoproduct formation, mainly pyrimidine dimers, which has been shown to lead to skin cancer in animal models [12]. Moreover, malignant melanoma is predominantly caused by UVA radiation via the indirect DNA damage, such as 8-oxo-deoxyguanosine, caused by free radicals and ROS [13]. UVB phototherapy has also been found to be able to induce ROS, and this has been suggested to be a major contributor to photocarcinogenesis, conditioning skin cell mutagenesis, tissue remodeling, inflammation, and immunosuppression [14]. Therefore, the abundance of NB-UVB generation of ROS in comparison to BB-UVB, as well as the long-term effects of UVB-induced indirect and direct DNA damage, is an area of great interest. Currently, long-term side effects of NB-UVB phototherapy are still unclear and under investigation [15,16]. To date, no clear evidence of NB-UVB phototherapy-induced human skin cancer has been reported.

The role of NB-UVB seems to be less clear in the management of skin conditions beyond psoriasis, as well as its potential risks. A prior study with mice reported that NB-UVB causes more mutations in p53 than BB-UVB at minimum erythema dosages (MED) [17]. MED values are related to repair of photolesions in transcriptionally active genes. The typical MED value in humans for NB-UVB ranges between 5000 J/m^2^ and 10,000 J/m^2^, which varies with race, skin type, and device used [18]. In addition, UVB phototherapy may induce short-term side effects on the skin, including sunburn-like erythema, xerosis accompanied by pruritus, occasional blistering, and an increased frequency of recurrent herpes simplex viral infections [19]. A serious concern utilizing UVB in phototherapy comes from UVR exposure being a proven carcinogen and causing various types of skin cancer. The most common form of skin cancer is basal-cell carcinoma (BCC), followed by squamous cell carcinoma (SCC), both of which often carry a UV signature mutation, indicating that these cancers are caused by UVB via direct DNA damage, forming CPDs [20]. Prior studies have concluded no significant association was found between patients treated with NB-UVB phototherapy and BCC, SCC, nor melanoma [15,16]. However, individuals with Xeroderma Pigmentosum (XP) syndrome are severely sensitive to UV light [21]. Such individuals are deficient in nucleotide excision repair (NER), important for proper repair to UVB-induced direct DNA damage, and have a high predisposition for skin cancers, including BCC, SCC, and melanoma. As a result, these individuals may still be at a high risk for developing such cancers following NB-UVB phototherapy [22].

In the present study, we examined the cytotoxicity via cell survival, genotoxicity via sister chromatid exchange (SCE) frequency, mutagenicity via hypoxanthine phosphoribosyltransferase (*HPRT*) mutation frequency, as well as CPD formation and ROS generation of NB-UVB in comparison to BB-UVB in mammalian CHO and their NER mutant cells. A prior study reported that NB-UVB cytotoxicity was induced at dosages 20 times lower than those required for BB-UVB-induced cytotoxicity [23]. To the best of our knowledge, no other studies have examined SCE or *HPRT* mutation frequencies following NB-UVB exposure. On the other hand, it has been demonstrated that BB-UVB exposure correlates linearly with SCE and *HPRT* mutation frequencies [24,25]. Therefore, we hypothesized this would be true for NB-UVB as well, but at dosages multiple times lower than required for BB-UVB.

Our results demonstrate that NB-UVB exposure is less harmful than BB-UVB, but still had toxic effects at higher cytotoxic dosages. This is of clinical importance as a prior study observed significantly better clinical outcomes after three months of high-dose NB-UVB therapy for psoriasis, with fewer required treatments, in comparison to low-dose regimens. Thus, they concluded NB-UVB phototherapy in a high-dose regimen for psoriasis should be recommended [26]. Results of this study will provide biological evidence for the use of NB-UVB over BB-UVB, and will aid in the selection of proper treatment dosages to help limit potential long-term health effects following NB-UVB phototherapy for the treatment of skin diseases.

## 2. Materials and Methods

### 2.1. Cell Culture and Conditions

Chinese hamster ovary (CHO) wild type CHO10B2 and XPG mutated UV-sensitive UV-135 [27] cells were kindly supplied by Dr. Joel Bedford (Colorado State University, Fort Collins, CO, USA). CHO cells were isolated in 1958 and hundreds of isogenic mutants are available. UV135 is one of the mutants which has the *XPG* gene mutated and is deficient for nucleotide excision repair pathway that manages UV-induced DNA damage [27,28]. Cells were grown and maintained in α-MEM medium (Invitrogen, Carlsbad, CA, USA) supplemented with 10% heat inactivated bovine serum (Sigma, St. Louis, MO, USA) (56 °C for 30 min), penicillin (100 units/mL), and streptomycin (100 μg/mL) in a humidified 5% CO_2_ atmosphere at 37 °C. The mean population doubling time for both cell lines was approximately 14 h.

Synchronization of cell population in the G1-phase of the cell cycle was accomplished by the mitotic shake off method [29]. Briefly, 10^5^ cells from stock cultures were seeded in plastic T-75 flasks. When growth of the cultures reached 50–80% confluence, the flasks were mechanically shaken and loosely attached mitotic cells were floated to culture medium and collected. Mitotic cells were subcultured to dishes and incubated for 2 h at 37 °C before UV exposure.

### 2.2. UV Light Exposure

Cells were plated onto P-30 polystyrene petri dishes (Greiner Bio-One, Frickenhausen, Germany) and washed with PBS twice. Subsequently, 1 mL of PBS was added to prevent dryness during UV exposure for experiments without petri dish caps. Cells were exposed to UV light sources at room temperature. Phillips germicidal UVC lamps (Phillips, Andover, MA, USA) were used for the UVC source, with a dose rate of 58.2 J/m^2^ per minute. Cell culture dishes were rotated at 8 rpm during UVC exposure. Six Westinghouse Sunlamps (Westinghouse, Cranberry Township, PA, USA) were used for the BB-UVB source, with a dose rate of 63.6 J/m^2^ per minute or 30 J/m^2^ per minute with or without the petri dish cap on during irradiation, respectively. A prior study observed that polystyrene will absorb wavelengths no greater than 300 nm; therefore, the polystyrene petri dish cap was either on or off during irradiation to address filtration of the BB-UVB light (280–315 nm) [30]. One 40 W Phillips TL01 lamp (Phillips) was used for NB-UVB source, with a dose rate of 82.8 J/m^2^ per minute. Dosimetry was carried out using a UVP UVX dosimeter with UVC and UVB probes (UVP, Upland, CA, USA). Spectra of each light were obtained using a ASEQ-LR1 spectroradiometer (ASEQ instruments, Vancouver, BC, Canada).

### 2.3. Cell Survival Analysis

Survival curves were obtained by a standard colony formation assay as previously described [31]. Immediately after exposure to UV light, cells were trypsinized. After trypsinization, the cells were plated in an appropriate concentration onto 60 mm plastic tissue culture dishes and returned to the incubator for seven days. The cultures were fixed with absolute ethanol and stained with 0.1% crystal violet. Lastly, colonies containing more than 50 cells were scored as survivors [32,33].

### 2.4. Sister Chromatid Exchange Assay

CHO synchronized in G1 phase cells were subcultured into four P-60 petri dishes at a density of approximately 10^6^ cells per dish in medium containing 5-bromo-2′-deoxyuridine (BrdU) (Sigma), with a final concentration of 10 μM for two cycles. Then, 0.2 μg/mL colcemid (Gibco, Invitrogen, Grand Island, NY, USA) was added to replicate petri dishes for four-hour intervals beginning at 26 or 32 h for SCE, depending upon the peak number of second passage mitosis SCE were scored in cultures containing the peak number of second-passage mitosis. Cells were harvested during metaphase, trypsinized, and then suspended in 2 mL of 75 mM KCl solution warmed to 37 °C and placed in a 37 °C water bath for 20 min. A fixative solution of 3:1 methanol to acetic acid was added to the samples according to the standard protocol [34]. Fixed cells were dropped onto slides and allowed to dry at room temperature. Differential staining of metaphase chromosomes was completed using the fluorescence plus Giemsa (FPG) technique with Hoechst 33,258 dye, followed by UVR exposure [35]. Differentially stained metaphase chromosome images were scored under a Zeiss Axioskop microscope equipped with a SPOT CCD camera RT 2.3.1 (Diagnostic Instrument, Inc., Sterling Heights, MI, USA) and SPOT basic software. A minimum of 50 metaphase cells were scored for each experiment. Data presented are the mean of SCE frequency per chromosome.

### 2.5. Mutagenicity Assay

The *HPRT* gene is on the X chromosome of mammalian cells, and is used as a model gene to investigate DNA damaging agents that lead to gene mutations [36]. 6-thioguanine (6TG) and other purine analogues select for *HPRT* mutant (-) cells, normal cells will die by taking up 6TG. The *HPRT* gene product is one of the many enzymes responsible for the salvage of preformed purine bases during normal nucleic acid turnover in mammalian cells [37]. The *HPRT* gene of CHO cells is functionally hemizygous in pseudodiploid cells since one X-chromosome is inactivated in female cells during embryonic development [38]. The Chinese hamster *HPRT* gene is located at the distal end of the short arm of the X chromosome where DNA replication occurs in early S phase. In this study, prior to use in the mutation assay, cells were grown in HAT medium (Sigma) for three days to reduce the level of spontaneous *HPRT* mutants. HAT is complete medium with 2 × 10^−4^ M hypoxanthine, 2 × 10^−7^ M aminopterin and 1.75 × 10^−5^ M thymidine. Cells for mutation studies were used within several days after HAT treatment. After UVR irradiation, G1 phase cultures were diluted and grown in nonselective medium for a period of time sufficient to allow phenotypic expression prior to plating for determination of mutant fraction. Phenotypic expression time is 7–10 days for *HPRT* mutation [39]. After seven days of phenotypic expression, cells were plated in petri dishes in the presence of a selective agent (5 μg/mL of 6TG) (Sigma). Cells from each culture were plated at a low density in the absence of the selective agent to determine plating efficiency. All cultures were incubated 10–12 days prior to scoring colonies. Colonies were fixed and stained as described in our colony formation assay. Colonies containing more than 50 cells were scored as survivors.

### 2.6. CPD Formation Assay

In total, 400,000 CHO10B2 cells were plated into each P-35 petri dish (Falcon, Oxnard, CA, USA) and incubated at 37 °C in a humidified 5% CO_2_ atmosphere incubator the night before UV light exposure. Following exposure PBS was removed and DNA extraction was done. Extraction buffer (10 mM Tris-HCl, 400 mM NaCl, 2 mM EDTA), 10% Sodium dodecyl sulfate (SDS), and 0.1 mg/mL Proteinase K (Roche Diagnostics Corporation, Indianapolis, IN, USA) was added and left in 37 °C incubator overnight. Next, 6 M NaCl was added and mixture was centrifuged at 2500 rpm for 15 min followed by transferring the supernatant to 70% EtOH and centrifuged again at 1000 rpm for 5 min. Supernatant was then discarded and contents were left to dry at room temperature. TE buffer (10 mM Tris and 1 mM EDTA) was used to dissolve contents and DNA concentration was measured via NanoDrop ND-1000 Spectrophotometer (NanoDrop Technologies, Wilmington, DE, USA) and stored at 4 °C. 100 μL of each experimental DNA diluted in PBS (5 μg/mL) was added to each well in the Falcon Microtest III 96 U-bottom well assay plate (Becton Dickinson and Co., Oxnard, CA USA) after 95 °C denature for 15 min and incubated overnight at 37 °C. Afterwards, an ELISA procedure was carried out as follows: each well was washed with PBS twice, and 10% goat serum in PBS was added to each well and incubated at 37 °C for 30 min followed by PBS wash twice, incubated for 1 h with 1:1000 primary antibody monoclonal anti-thymine dimer antibody produced in mouse (Sigma) in 10% goat serum, washed twice with PBST (0.05% Tween 20 in PBS) and twice with PBS, incubated for 1 h with 1:3000 secondary antibody Alexa Fluor 488 goat anti-mouse IgG (Invitrogen Molecular Probes, Eugene, OR USA) in 10% goat serum, washed with PBST and PBS as before. Next, TACS-Sapphire (Trevigen, Gaithersburg, MD, USA) was added to each well and allowed to incubate for 15 min at room temperature protected from light; blue color indicated a positive result. Then, 0.2 M HCl was added to each well and fluorescence of each well was addressed in Bio-Rad Benchmark Microplate Reader (Bio-Rad laboratories, Hercules, CA USA) read at 450 nm with reference of 595 nm. The absorbance at 450 nm was positively proportional to the concentration of thymine dimer.

### 2.7. Detection of Reactive Oxygen Species

ROS was observed utilizing the oxidative stress indicator, Carboxy-H_2_DCFDA, which is a nonfluorescent reagent. Carboxy-H_2_DCFDA is oxidized in the presence of ROS and will then fluoresce green. Therefore, amount of fluorescence is directly proportional to the amount of ROS generated following each UVR exposure. Suspension of 100,000 CHO cells in 1 mL PBS were placed into their respective P35 petri dish (Falcon) and irradiated with NB-UVB, BB-UVB, and UVC for 1 h with the petri dish cap off. Negative control cells were not exposed to UVR and shielded from light with aluminum foil. Following exposure, all cells were transferred to their respective centrifuge tube, treated with oxidative stress indicator, 25 μM Carboxy-H_2_DCFDA (Life Technologies, Eugene, OR, USA). After treatment, samples were incubated for 1 h at room temperature shielded from light. Following incubation, 5 mL PBS was added, and cells were centrifuged at 1000 rpm for 5 min. Supernatant was discarded to remove excess Carboxy-H_2_DCFDA. Pelleted cells were resuspended in 1 mL PBS and fluorescent signal was obtained with VersaFluor^TM^ Fluorometer (Bio-Rad) and was blanked with a negative control. Negative control fluorescence was recorded again following the collection of fluorescent data of all other sets in the experiment to account for background fluorescence.

### 2.8. Statistical Analysis

All experiments were carried out with three or more independent experiments. Data points were expressed as a mean with standard errors of the means. All experimental data were analyzed via Prism 5^TM^ software (GraphPad, La Jolla, CA, USA). One-way analysis of variance (ANOVA) and Tukey’s multiple comparison test was conducted for statistical significance. Differences with *p* values of less than 0.05 were considered statistically significant.

## 3. Results

### 3.1. UV Spectrum

**Figure 1:**
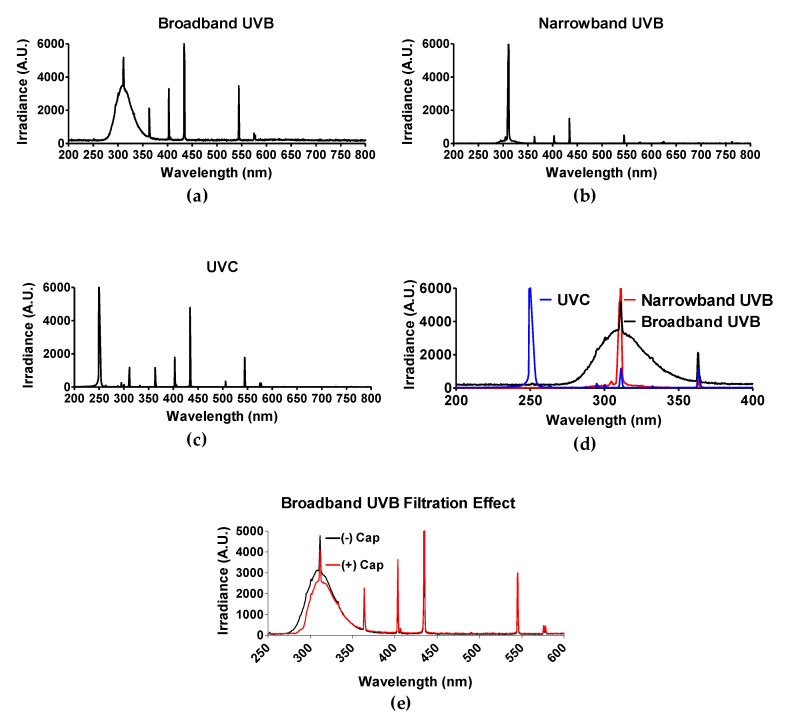
Spectrum of each UV light source. Three light devices were used in this study: (**a**) Broadband UVB-Westinghouse Sunlamp (Broad spectrum 280–350 nm, 63.6 J/m^2^ per minute); (**b**) Narrowband UVB-Phillips TL01 (311 nm peak, 82.8 J/m^2^ per minute); (**c**) UVC-germicidal lamp (254 nm peak, 58.2 J/m^2^ per minute). (**d**) Merge of UV spectrum. Light spectrum was confirmed with spectroradiometer Aseq-LR1. (**e**) Filtration effect of petri dish cap either on (+) or off (−) with conventional broadband ultraviolet-B (BB-UVB) irradiation. The dose rate (J/m^2^) of each light device was measured with a UVP-UVX radiometer with UVC and UVB probes.

The light spectrum of each UV light source was obtained by spectroradiometer Aseq-LR1 and is illustrated in Figure 1d. As mentioned in the materials and methods section, polystyrene will absorb wavelengths no greater than 300 nm [30]. Therefore, only experiments utilizing BB-UVB (280–315 nm) were addressed for the filtration effect of having the polystyrene petri dish cap on or off during irradiation, while experiments utilizing UVC (100–280 nm) and NB-UVB (e.g., 311 nm monochromatic) were irradiated with the polystyrene petri dish cap off during irradiation. BB-UVB without petri dish cap on during irradiation displayed a peak at a wavelength of 311 nm with an irradiance of 5191.15 arbitrary unit (A.U.) and 50% of the irradiance (2595.58 A.U.) at wavelengths below 298 nm and above 322 nm, and 25% of the irradiance (1297.78 A.U.) falling below 289 nm and above 336 nm (Figure 1a). BB-UVB having the polystyrene petri dish cap on during irradiation demonstrated to filter some of the wavelengths within the BB-UVB spectrum (280–315 nm). BB-UVB with the cap on displayed a peak at a wavelength of 311 nm with an irradiance of 4118.36 A.U. and 50% of the irradiance (2059.18 A.U.) at wavelengths below 302 nm and above 324 nm, and 25% of the irradiance (1029.59 A.U.) falling below 295 nm and above 337 nm (Figure 1e). NB-UVB displayed a sharp peak at 311.59 nm of 6910.05 A.U. with 50% of the irradiance (3455.03 A.U.) at wavelengths below 309.59 nm and above 311.72 nm, and 25% of the irradiance (1727.51 A.U.) at wavelengths below 309.06 nm and above 312.00 nm (Figure 1b). UVC produced a peak from wavelengths 250–252 nm, all containing an irradiance of 16,383 A.U. 50% of the irradiance (8191.50 A.U.) at wavelengths below 249 nm and above 253 nm, and 25% of the irradiance (4095.75 A.U.) at wavelengths below 247 nm and above 254 nm (Figure 1c).

### 3.2. Cytotoxicity of Narrowband UVB

**Figure 2:**
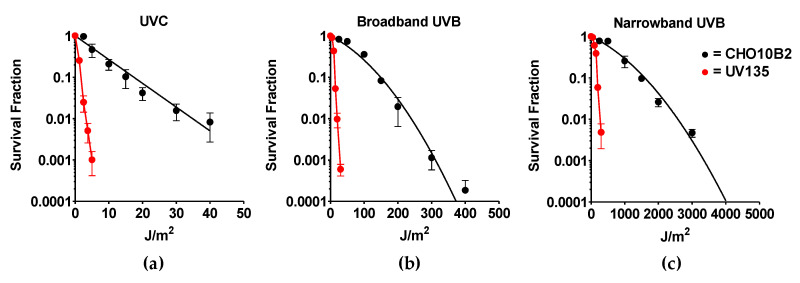
UV exposure cell survival curves. (**a**) Ultraviolet C (UVC)-exposed cells; (**b**) Broadband UVB (BB-UVB)-exposed cells; (**c**) Narrowband UVB (NB-UVB)-exposed cells. Black circles indicate wildtype CHO10B2 cells. Red circles indicate XPG mutant UV135 cells. Error bars indicate standard errors of the means from as many as three independent experiments.

Exponentially growing CHO10B2 (wild type) and UV135 (XPG mutant) cells were exposed to UVC, BB-UVB, and NB-UVB. Cytotoxic effects were tested by colony formation assay. The dose response for cell survival curve is shown in Figure 2. The D_10_ value, the dose to kill 90% of the cell population, revealed the killing efficiency per absorbed dose to be 17 J/m^2^ for UVC, 149 J/m^2^ for BB-UVB, and 1596 J/m^2^ for NB-UVB for CHO10B2 cells (Table 1). Our data show that UVC killed cells the most efficiently, while BB-UVB killed more efficiently than NB-UVB per absorbed dose (J/m^2^).

Our results also portrayed severe sensitivity to all types of UVR in UV135 cells (Figure 2). Almost 10 times higher sensitivity was calculated from D_10_ values for each UV exposure; 1.7 J/m^2^ for UVC, 14 J/m^2^ for BB-UVB, and 197 J/m^2^ for NB-UVB (Table 1). This increased sensitivity of the XPG mutant UV135 cells demonstrates the ability of NB-UVB to create DNA damage repaired by nucleotide excision repair.

### 3.3. Genotoxicity and Mutagenicity of Narrowband UVB

**Figure 3:**
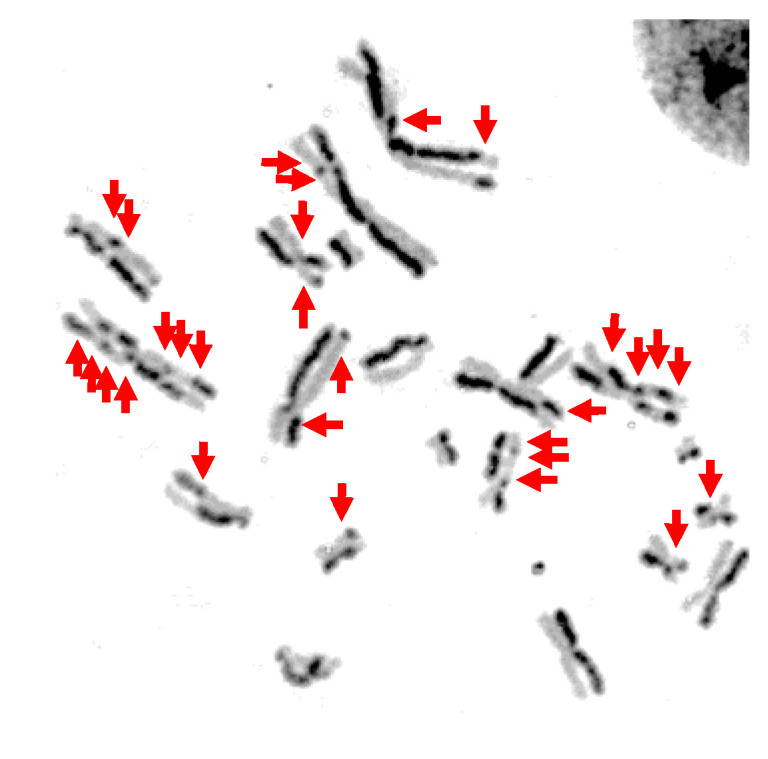
Representative image of sister chromatid exchange (SCE) after NB-UVB 500 J/m^2^: Red arrows indicate sister chromatid exchanges.

**Figure 4:**
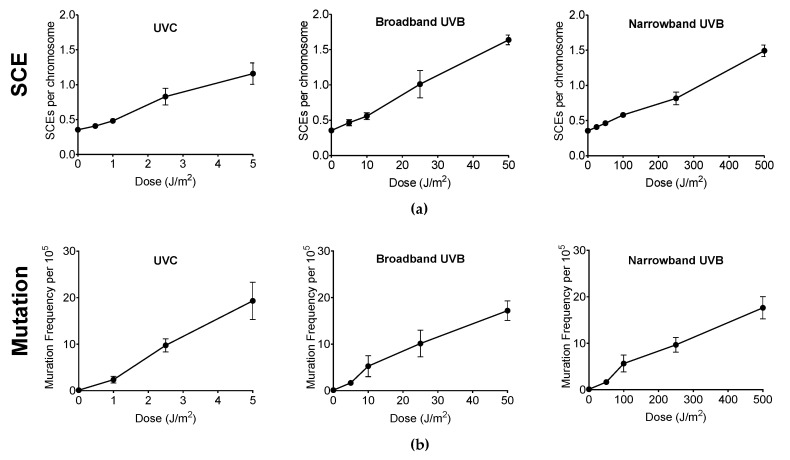
Genotoxicity and mutagenicity following UV exposure in CHO10B2 cells. (**a**) Sister chromatid exchange (SCE) frequency following UVC, BB-UVB, and NB-UVB at increasing dosage. (**b**) Hypoxanthine phosphoribosyltransferase (*HPRT*) mutation frequency following UVC, BB-UVB, and NB-UVB at increasing dosage. Error bars indicate standard errors of the means from at least three independent experiments.

Genotoxicity and mutagenicity of NB-UVB was examined via DNA replication genotoxic stress quantitatively evaluated by sister chromatid exchange (SCE) (Figure 3) and hypoxanthine phosphoribosyltransferase (HPRT) mutation frequency. The dose–response curves for the induction of SCE and induction of 6TG resistance (6TG^r^) mutation by various UVR exposure in G1-phase synchronized CHO10B2 cells is shown in Figure 4a,b, respectively. Tested doses were noncytotoxic doses, which were obtained from the prior cell survival curves (Figure 2). The frequencies of both SCE and HPRT mutation were linearly elevated up to 5 J/m^2^ with 0.17 SCE per J/m^2^ and with 2.3 HPRT mutation per 10^5^ cells per J/m^2^ for UVC, 50 J/m^2^ with 0.02 SCE per J/m^2^ with 0.35 HPRT mutation per 10^5^ cells per J/m^2^ for BB-UVB, and 500 J/m^2^ with 0.002 SCE per J/m^2^ with 0.039 HPRT mutation per 10^5^ cells per J/m^2^ for NB-UVB. SCEs per chromosome mean values were 1.16 for 5 J/m^2^ dose of UVC, 1.64 for 50 J/m^2^ dose of BB-UVB, and 1.49 for 500 J/m^2^ dose of NB-UVB. HPRT mutation frequencies per 10^5^ cells mean values at each of these respective dosages were 19.31 for 5 J/m^2^ dose of UVC, 17.20 for 50 J/m^2^ dose of BB-UVB, and 17.65 for 500 J/m^2^ dose of NB-UVB. These results indicate that NB-UVB can induce genotoxic and mutagenetic effects even at low noncytotoxic dosages, and demonstrate that NB-UVB survivors carried base damages into S-phase after G1-phase irradiation.

### 3.4. Cyclobutane Pyrimidine Dimer (CPD) Formation of Narrowband UVB

**Figure 5:**
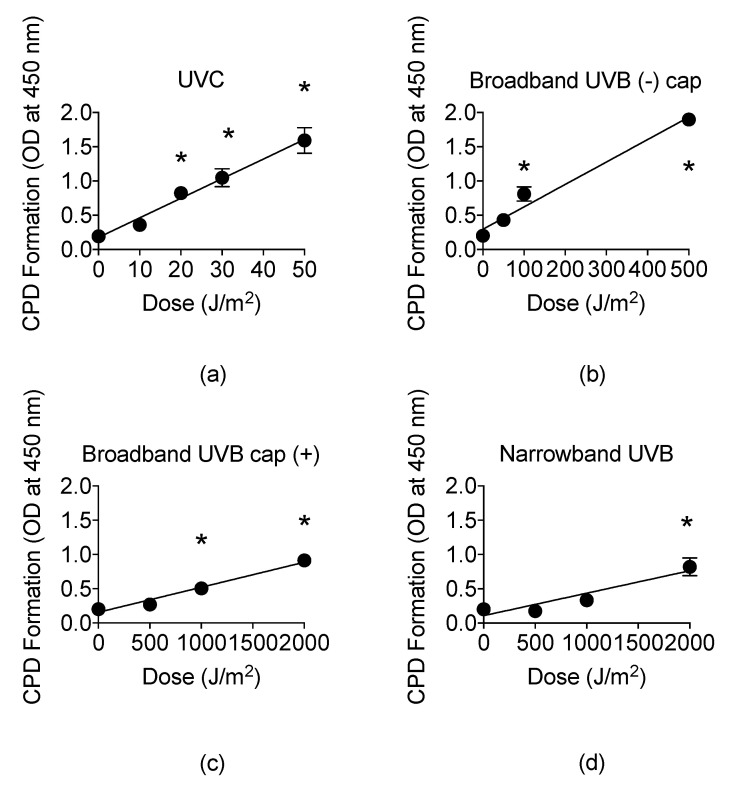
UV light formation of cyclobutane pyrimidine dimers (CPDs) in CHO10B2 cells at noncytotoxic and cytotoxic dosages. (**a**) UVC-exposed cells; (**b**) Broadband-UVB-exposed cells without petri dish cap off during exposure; (**c**) Broadband-UVB-exposed cells with petri dish cap on during exposure; (**d**) Narrowband-UVB-exposed cells. Error bars indicate standard error of the mean of at least three independent experiments. * Indicates statistically significant differences compared to control (*p* < 0.05), one-way ANOVA followed by Turkey’s Multiple Comparison Test.

The presence of CPDs is unique to UVR and identifies UVR as a mutagen [9]. Therefore, we examined the CPD formation by ELISA assay, ranging from noncytotoxic to cytotoxic doses obtained from the cell survival curves for each type of UVR exposure. For UVC, CPD formation linearly increased at a rate of 0.029 per J/m^2^ with a mean value of 1.59 at 50 J/m^2^ (Figure 5a). The CPD formation linearly increased up to 500 J/m^2^ at a rate of 0.0033 per J/m^2^ for BB-UVB without the petri dish cap, 2000 J/m^2^ for BB-UVB with the petri dish cap, and 2000 J/m^2^ at a rate of 0.00033 per J/m^2^ for NB-UVB (Figure 5b–d).

A higher dosage was observed to be required in order to induce a significant increase in CPD formation for BB-UVB with the cap on in comparison to BB-UVB with the cap off during irradiation, with a mean value of 1.86 at 500 J/m^2^ and 0.91 at 2000 J/m^2^, respectively (*p* < 0.05) (Figure 5b,c). Moreover, BB-UVB exposure of samples with the petri dish cap on followed a similar linear increase in CPD formation trend as NB-UVB exposure, although a lower dosage was required for this trend (Figure 5d). Furthermore, NB-UVB required the highest dosage, 2000 J/m^2^, in order to induce a significant increase in CPDs with a mean value of 0.69 (*p* < 0.05) (Figure 5d). These results show that UVB induces CPDs in a dose-dependent manner, and NB-UVB requires higher dosages than BB-UVB to produce a significant increase in CPDs.

### 3.5. ROS Production of Narrowband UVB

**Figure 6:**
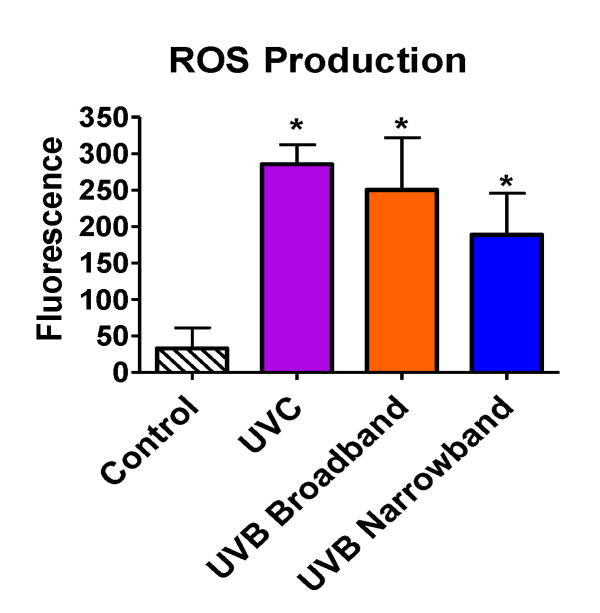
Oxidative stress following 1 h of UV light exposure of CHO10B2 cells. Error bars indicate standard error of the mean of at least three independent experiments. * Indicates statistically significant differences compared to control (*p* < 0.05), one-way ANOVA followed by Turkey’s Multiple Comparison Test.

Beyond the ability of NB-UVB to cause direct DNA damage via formation of CPDs, we tested the capability of NB-UVB to induce indirect DNA damage via production of ROS by ROS detection assay. All samples were exposed with the appropriate UVR for 1 h. Exposure dosages for UVC, BB-UVB, and NB-UVB were 3492 J/m^2^, 3816 J/m^2^, and 4968 J/m^2^, respectively. Our results portrayed a significant increase in ROS production inside the cells for UVC, BB-UVB, and NB-UVB, with mean fluorescence values of 285.5, 250.25, and 188.75, respectively (*p* < 0.05) (Figure 6). While BB-UVB more efficiently produced ROS per absorbed dosage (J/m^2^), it was not found to be significant in comparison to NB-UVB (*p* > 0.05). It is important to note that although this dosage of UVC is very lethal, a significant increase of ROS production was still observed. These results suggest that while the main mechanism for NB-UVB to induce DNA damage may be directly through formation of CPDs, NB-UVB can still produce a significant increase in ROS at higher cytotoxic dosages.

### 3.6. CPD Formation in Comparison to Cytotoxicity and Genotoxicity

**Figure 7:**
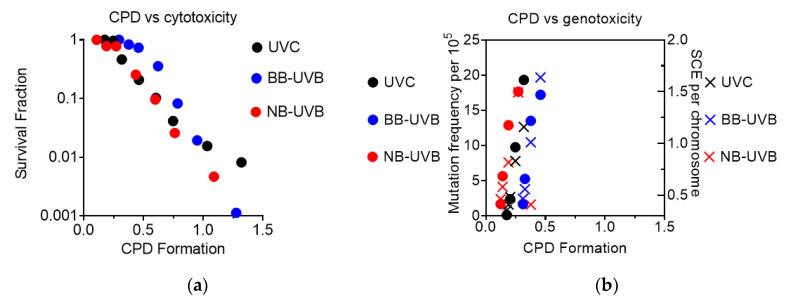
Summary of CPD formation vs. cytotoxicity and genotoxicity. (**a**) CPD formation vs. cytotoxicity; (**b**) CPD formation vs. genotoxicity.

CPD formation was observed to correlate linearly with cytotoxicity, as the increase in CPD formation led to a decrease in the survival fraction under all irradiation conditions (Figure 7a). It was also observed that genotoxicity followed a similar trend, as increases at low levels of CPD formation led to an increase in both mutation and SCE frequency under all irradiation conditions (Figure 7b).

## 4. Discussion

Prior in vivo studies have considered that NB-UVB had greater carcinogenic potential for DNA damage than BB-UVB at equal erythemal doses of 1 MED with a dose of 8500 J/m^2^ or 3700 J/m^2^ and 2500 J/m^2^ or 1700 J/m^2^ for NB-UVB and BB-UVB in C5BL/6J mice or albino hairless mice, respectively [17,40]. However, at minimal cytotoxic doses, the reverse has been observed [23]. Knowledge of the biological effects of NB-UVB at various dosages is vital for developing protection standards for occupational, medical, and clinical exposure. In addition, the type of UVR may influence the mechanism in which exposure causes DNA damage. UVB is known to cause direct DNA damage via the production of CPDs, and UVA is known to cause indirect DNA damage through the production of ROS, as well as inducing photodimers. Several studies have shown UVB can induce ROS, including superoxide radicals, hydrogen peroxide, and hydroxy radicals [41,42]. One of these studies showed that UVB light, in the range of 10–1000 J/m^2^, caused a marked increase in the formation of ROS in both human and mouse keratinocytes [42]. Additionally, previous research showed ROS scavengers can reduce the cytotoxicity of UVB [43,44]. Our results agree with these prior studies of ROS generated by UVB, as we observed that both BB-UVB and NB-UVB were capable of inducing ROS. Moreover, we demonstrated that BB-UVB more efficiently produced ROS than NB-UVB per absorbed dose (J/m^2^) (Figure 6). Importantly, at a high cytotoxic dosage, we observed that NB-UVB can significantly induce ROS. These results suggest that NB-UVB at high cytotoxic dosages may induce DNA damage both directly and indirectly.

In the present study, NB-UVB was observed to be toxic at high doses, but less toxic than both BB-UVB and UVC in wildtype and UV-sensitive mutant CHO mammalian cell lines. The results from the colony formation assay demonstrated UVC to be the most cytotoxic, followed by BB-UVB and then NB-UVB per absorbed dose (J/m^2^) (Figure 2). D_10_ values calculated from these survival curves revealed that NB-UVB was nearly 100× less cytotoxic than UVC and 10× less cytotoxic than BB-UVB (Table 1). Our results confirmed and extended findings from a prior study that observed that NB-UVB cytotoxicity required at least 10 times the dosage for cytotoxicity with BB-UVB in human cell lines [23]. We also observed an increase of cytotoxicity in our UV135 cell line following NB-UVB exposure. Thus, our results demonstrated the ability of NB-UVB to create DNA damage repaired by NER (Table 1). However, UVC- and BB-UVB-exposed cells were nearly 10 times more sensitive than wildtype CHO10B2 cells, while NB-UVB exposed cells were around 8 times more sensitive. This might have been because some of the DNA damage induced by NB-UVB may have been repaired by another mechanism other than NER.

To further investigate the potential of NB-UVB to induce cancer, we observed the genotoxicity and mutagenicity following UVR exposure in G1-phase synchronized cells at noncytotoxic dosages, as determined from our colony formation assays. Genotoxicity of UVR was addressed by the SCE assay as SCEs have been interpreted as a measure of lesions in G1 and repaired at a later cell cycle stage when the sister chromatid becomes available (mid S–G2) following exposure to a mutagen, UVR in our case [45,46]. Most SCEs appear to arise from long-lived residual base damage and DNA–DNA or DNA–protein cross-links, which cause a significant distortion in the DNA double helix and replication fork stall [47,48]. Errors such as misincorporation or base mismatches that may occur during S-phase would lead to the induction of SCE from homology-directed DNA double strand break repair. A prior study demonstrated UVC light exposure to G_1_ synchronized CHO and V79 cells induced SCEs caused by the formation of persistent lesions that lead to exchanges only if the lesion-bearing chromosomes pass through S-phase [49]. Our results agree with this prior study, as we observed SCE frequency to increase following UVC exposure. Moreover, the observation that SCE frequency increased following NB-UVB exposure demonstrates that these colonies carried base damages into S-phase after G1-phase irradiation. Other studies have demonstrated SCE frequency increases with UVR dose. One such study observed SCE frequency to correlate linearly with UVC and P-UVA exposure in V79 Chinese hamster cells [50], and another study observed SCE frequency to correlate linearly with BB-UVB exposure in the telomeres of CHO cells [24]. Our results agree with these prior studies, as we observed the frequency of SCE to linearly elevate for UVC and BB-UVB. In addition, we also observed the SCE frequency to correlate linearly following NB-UVB exposure (Figure 4a). However, our results demonstrated that NB-UVB was approximately 10 times less efficient for the induction of SCEs than BB-UVB per absorbed dose (J/m^2^).

Mutagenicity of NB-UVB exposure was then addressed by the *HPRT* mutation assay. It has been demonstrated that BB-UVB is capable of producing mutations via the *HPRT* mutation assay in V79 Chinese hamster and GM02359-hTERT cell lines [25,51]. Our results further these findings, as the dose–response curve for the induction of 6TG^r^ mutation (*HPRT* mutation frequency) was linearly elevated for NB-UVB in CHO10B2 cells (Figure 4b). A prior study using *Ogg1* knockout mice demonstrated that NB-UVB induces more p53 mutations than BB-UVB at 1 MED, 456 J/m^2^/min and 228 J/m^2^/min for NB-UVB and BB-UVB, respectively [17]. However, our results demonstrated an approximate 10-fold reduction of *HPRT* mutation frequencies with NB-UVB in comparison to BB-UVB per absorbed dose (J/m^2^).

Several investigators have reported that mean SCE frequencies correlate directly with mutation frequencies induced by the class of chemical agents that induced primarily DNA base damage [52,53,54,55]. Interestingly, we observed that the trend of SCE and *HPRT* mutation induction was very similar in the noncytotoxic dosages of UVC, BB-UVB, and NB-UVB exposure (Figure 4a,b). It is important to note that even though this trend was similar, NB-UVB required 10 times the dosage required to induce these effects than BB-UVB. In addition, it was observed that both SCE and *HPRT* mutation frequency increased at dosages less than 100 J/m^2^ of NB-UVB exposure, which is a noncytotoxic dose. Importantly, NB-UVB induced SCE and *HPRT* mutations are a novel finding in this study. Though the mechanism remains unclear, the fact that cytogenetic changes in a clinically relevant but nonlethal UVB doses can be induced offers further evidence of the risk for the exposed individuals. These findings may also have important implications for exposure standards for the medical occupational individuals and patients.

The presence of CC → TT base changes is known to identify UVB as a mutagen, as they are only known to be caused by the direct absorption of UVB energy on DNA, and the appearance of C → T transitions occur exclusively at dipyrimidines, which is also unique to UVR [9]. This is of clinical importance as both basal-cell carcinoma (BCC) and squamous cell carcinoma (SCC) often contain the mutation signature of CPD repair, which indicates that these are caused by UVB exposure. Multiple clinical studies have concluded that long-term exposure to NB-UVB is not associated with an increased risk of skin cancers such as melanoma, BCC, and SCC [15,16,56]. However, our results showed a linear increase in CPD formation following exposure to UVC, BB-UVB, and NB-UVB at dosages ranging from noncytotoxic to cytotoxic, as determined from the colony formation assay (Figure 5a–d). We observed significant increases in CPD formation at high cytotoxic dosages for each UV exposure (*p* < 0.05) (Figure 5a–d). It is important to note that these experiments were conducted in Chinese hamster ovary (CHO) cell lines and not human cell lines. This is of importance as CHO cells selectively remove CPD from transcriptionally active genes, making the genome wide repair of CPD very poor in contrast to pyrimidine-pyrimidone photoproducts (6-4PPs), which are repaired quickly and efficiently from the genome. On the other hand, human cells are known to repair both CPD and 6-4PPs efficiently [57]. Interestingly, we observed that BB-UVB more efficiently formed CPDs with the petri dish cap off per absorbed dose (J/m^2^), and that the CPD formation trend of BB-UVB with the cap on was similar to that of NB-UVB. Our results demonstrated NB-UVB was far less efficient at formation of CPDs in comparison to unfiltered BB-UVB per absorbed dose (J/m^2^) even when the cap was on during BB-UVB irradiation. In a prior study, Maeda et al. demonstrated the filtration effect of the petri dish cap being on during BB-UVB exposure [43]. Thus, we suggest that this observation may have been the result of shorter wavelengths within the BB-UVB spectrum not being able to penetrate the petri dish cap, essentially filtering the BB-UVB, which may explain the linear trend resembling that of NB-UVB.

These results demonstrate that NB-UVB is safer than BB-UVB in the aspect of CPD formation. However, it is of importance to address that NB-UVB phototherapy is administered in dosages that exceed the highest in vitro cytotoxic dosage used in our experiment, as the MED ranges between 5000 J/m^2^ and 10,000 J/m^2^, which varies with race, skin type, and device used [18]. It has been suggested that NB-UVB phototherapy should be given at very high treatment dosages, as a prior study revealed a significantly better clinical outcome following three months of this method of therapy. In that study, patients with Fitzpatrick skin phototype I and II were administered doses as high as 12,000 J/m^2^, and for patients with skin phototype III and IV, they administered doses as high as 15,000 J/m^2^ [26]. However, the long-term effects of this type of high dose NB-UVB phototherapy are not known. Our results indicate that at cytotoxic dosages of NB-UVB, far lower than the dosages used in that study, we were able to observe a significant increase in CPD formation (Figure 5d). Therefore, careful consideration should be given regarding the appropriate treatment dosage of NB-UVB, as such high dosages may provide a better clinical outcome for such skin diseases as psoriasis, but may lead to far worse long-term outcomes, such as BCC or SCC.

## 5. Conclusions

NB-UVB requires a much higher dosage than BB-UVB to induce in vitro toxic biological effects. Thus, results of this study indicate NB-UVB to be less toxic than BB-UVB per absorbed dose (J/m^2^), and that NB-UVB not only acts directly via the production of CPDs, but can also induce DNA damage indirectly through the production of ROS. In addition, growing interest in high dose NB-UVB phototherapy regimens should be carefully considered, as our results demonstrate significant increases in genotoxicity and mutagenicity at in vitro noncytotoxic doses of NB-UVB and CPD formation and ROS production at high in vitro cytotoxic doses of NB-UVB. Further studies in post-NB-UVB phototherapy patient analysis and in vivo research should be conducted to compare clinically relevant low and high dose long-term effects, as well as in comparison to the long-term effects induced by BB-UVB to further understand which of these therapies is best to use.

## Figures and Tables

**Table 1:** D_10_ values (dose required to kill 90% of cells) of ultraviolet radiation (UVR).

UVR Source	D_10_ Value by Cell Type (J/m^2^)	
	CHO10B2 Wild type	UV135 XPG mutant
UVC	17	1.7
Broadband UVB	149	14
Narrowband UVB	1596	197
